# Peer Support to Enhance Social and Emotional Self-Management Following Acquired Brain Injury Rehabilitation: Design of a Pre–post Study With Process Evaluation

**DOI:** 10.3389/fneur.2021.647773

**Published:** 2021-07-29

**Authors:** Milou Baumgartner-Dupuits, Simone J. S. Sep, Jeanine Verbunt, Hans Bosma, Jacques van Eijk

**Affiliations:** ^1^Care and Public Health Research Institute (Caphri), Faculty of Health, Medicine and Life Sciences, University Maastricht, Maastricht, Netherlands; ^2^Department of Rehabilitation Medicine, Faculty of Health, Medicine and Life Sciences, University Maastricht, Maastricht, Netherlands; ^3^Department of Social Medicine, Faculty of Health, Medicine and Life Sciences, University Maastricht, Maastricht, Netherlands; ^4^Adelante, Centre of Expertise in Rehabilitation and Audiology, Hoensbroek, Netherlands

**Keywords:** self-management, SMS, peer support, rehabilitation, acquired brain injury, self-efficacy, follow-up

## Abstract

**Background:** Specialized rehabilitation following acquired brain injury provides intensive multidisciplinary treatment to individuals with complex disabilities for optimizing recovery and supporting a safe transition to the community. Post-specialist rehabilitation, patients and caregivers have reported a need for support. We present the design of an implementation study to evaluate a new self-management support service for individuals with acquired brain injury and their caregivers.

**Methods:** This is a pre–post intervention study with a mixed-method design. The study population comprises individuals aged ≥18 years with acquired brain injury living independently following specialized rehabilitation in the Southern part of the Netherlands. All participants receive a post-rehabilitation support service. The support service consists of several house visits by a peer support volunteer in the first weeks after specialized rehabilitation treatment. The peer support volunteers are trained according to an adapted version of the previously developed Self-Management Support (SMS) program. The SMS program is directed at improving social and emotional self-management. Patient outcomes are assessed by questionnaire pre-, directly post-, and 6 months post-intervention. The primary patient outcome measure is self-efficacy. Secondary outcomes are perceived autonomy, quality of life, and psychological well-being. A process evaluation will be performed to gain insight into barriers and facilitators for the implementation of peer-led SMS by combining both quantitative, questionnaire data and qualitative data derived from focus groups with peer supporters and patients. In a workshop with relevant stakeholders, possibilities for dissemination and sustainability will be explored.

**Discussion:** This paper describes the design of a practice-based study on feasibility, barriers, and facilitators to the implementation of a home-based, peer-led self-management support intervention for patients with acquired brain injury. We will quantitatively and qualitatively evaluate the change in relevant patient outcomes pre- and post-intervention and the barriers and facilitators related to the implementation of the intervention. Following a positive evaluation, the final stage of the study aims to facilitate deployment and utilization of the intervention.

## Introduction

Advances in acute and critical care management have increased the number of people surviving acquired brain injury. As a sudden, severe event, acquired brain injury can cause persistent, even life-long consequences for the patients' participation. This burden affects the daily life of survivors and their families. In the Netherlands, at current, an estimated 650,000 people and their families are dealing with the consequences of acquired brain injury, accounting for 25% of total healthcare costs, not to mention relevant social costs (1).

Specialized rehabilitation care provides intensive multidisciplinary care to individuals with complex disabilities following acquired brain injury to optimize their recovery after hospital admission and support a safe transition to the community (2, 3). Post-specialist rehabilitation, the intensity of formal care, treatment, and support is strongly reduced. The transition from inpatient or outpatient rehabilitation to living at home independently is considered difficult by many patients (4–6).

Previous studies revealed that there is a need for support of patients and their informal caregivers when returning home after rehabilitation, and a need for the potentially added value of hands-on experts, such as peers, in providing this support (4, 6–8). Peer supporters have an exclusive expertise that can be useful, because they have experienced these difficulties themselves. In addiction and mental healthcare services, peer supporters have proven to be beneficial to the patients' activation; patients become healthier and have a better quality of life (9).

Tailored self-management interventions are of crucial importance for patients and informal caregivers to further optimize the path of care toward independent living and are expected to be of value post-rehabilitation (10). A widely applied self-management strategy in healthcare developed by Lorig et al. (11) focuses on three self-management tasks: medical management, role management, and emotional management. Self-management is closely linked to self-efficacy, as self-efficacy reflects the person's confidence in the belief in their own abilities (12). We previously studied Lorig's self-management strategy in the primary care setting (13). This Self-Management Support program (SMS) includes principles of problem solving and cognitive behavioral change (13). Although nurse-led SMS effectively improves self-efficacy, daily functioning, and social participation in multiple chronic conditions, its implementation in practice turned out to be difficult (14). A challenge has been the often disease-focused context in which SMS has to be integrated. More recently, we performed a pilot study among people that reached out to the municipality for support with social participation, in which SMS was provided by trained volunteers; that appeared to be successful (15). These volunteers showed that they could perform the intervention as well, in contrast to what is often expected by healthcare professionals (15).

The study presented here will focus on patients with acquired brain injury. Typically, an acquired brain injury is not hereditary; it is congenital, degenerative, or induced by birth trauma. Essentially, this type of brain injury occurs suddenly, leaving those who are severely affected struggling with everyday function and adaptation and facing challenges in everyday functioning (16). Improving these patients' problem solving skills and acquiring an active coping style are known to improve their health-related quality of life (3). Indeed, self-management interventions carried out by peer supporters have been successful (7, 17, 18). Previous studies, however, did not take into account the problems of the transition following rehabilitation, in the path of care toward living independent.

We present the design of a mixed-method pre–post study to evaluate the implementation of SMS provided by peer support volunteers (hereafter referred to as “peer supporters”) to adults with acquired brain injury following specialist rehabilitation in a large rehabilitation center in the South of The Netherlands. Besides the evaluation of patient outcome, a thorough process evaluation will be performed to investigate barriers and potential facilitators for the implementation of peer-led SMS for patients with acquired brain injury.

### Research Questions

#### Pre–post Evaluation

1. Does the degree of self-efficacy of individuals with brain injury improve after peer-led SMS?
2. Does self-efficacy improve more in patients who had a more optimal “dose” of peer-led SMS?

#### Process Evaluation

A process evaluation of the SMS intervention is directed toward the following research questions:

1. What are the reach, dose, and fidelity of the peer-led SMS intervention?
2. How is SMS experienced by patients, their informal caregivers, peer supporters, and health professionals?
3. What are barriers and facilitators of the peer-led SMS intervention?
4. How does the intended cooperation between health professionals and peer supporters evolve during their involvement with patients?
5. What is necessary for definitive implementation of the peer-led SMS intervention in the rehabilitation center?

#### Dissemination

In addition, implementation strategies for regional and national implementation will be explored:

1. What is, according to relevant stakeholders, necessary to successfully implement and disseminate cooperation between professionals and (volunteer) peer supporters?

## Methods/Design

### Study Design

**Figure 1:**
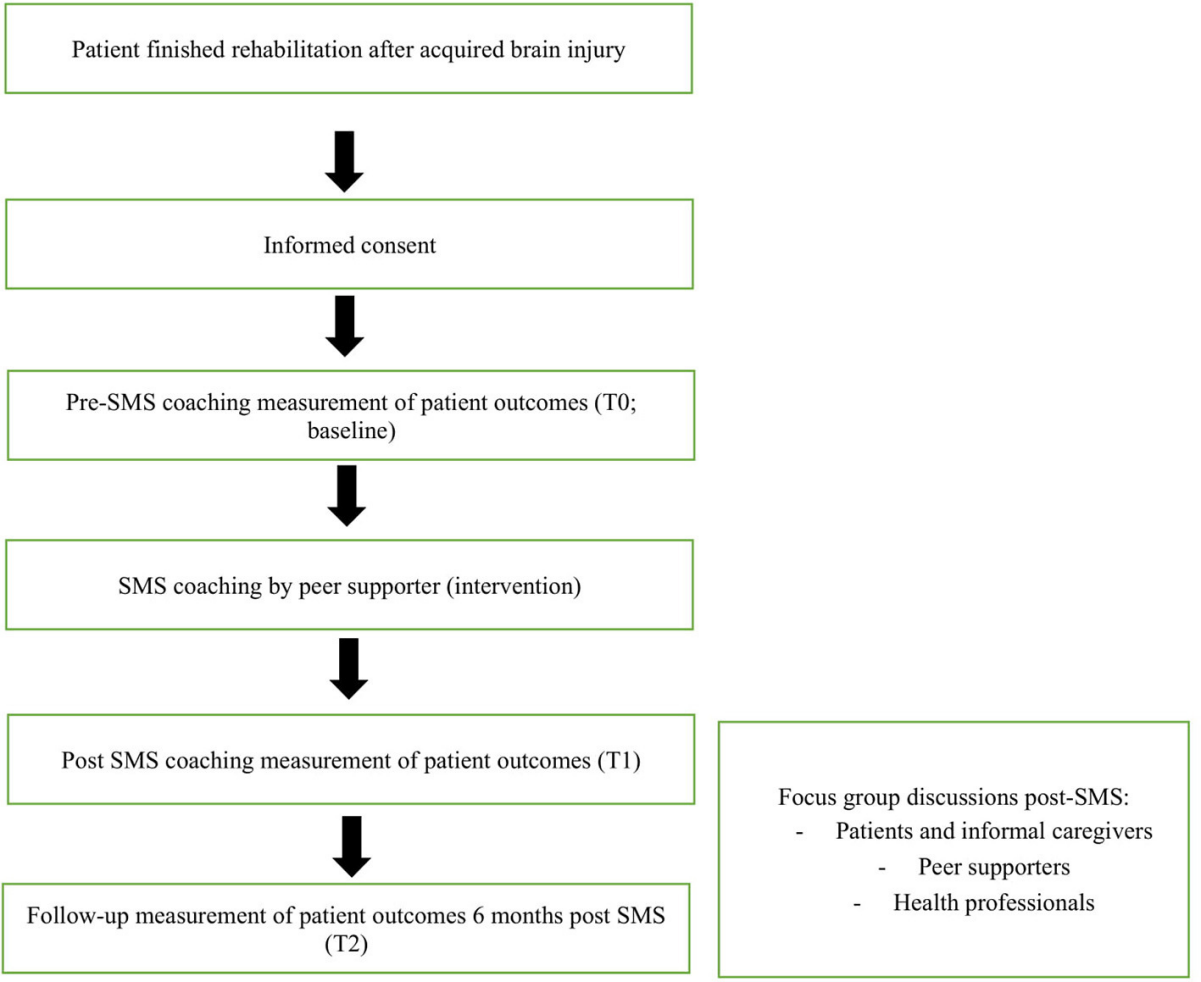
Schematic representation of the study design.

This is a pre–post intervention study with a mixed-method design. The peer-led SMS intervention starts 4 weeks after completion of the rehabilitation treatment at the patient's home. Participants will be followed up to 6 months post-intervention. Three questionnaire measurements will be conducted (pre-, directly post-, and 6 months post-intervention). In addition, in-depth qualitative data on the experiences with the intervention will be gathered by focus-group discussions post-intervention. A schematic representation of the study design is depicted in Figure 1. The study has been approved by the institutional Medical Ethics Committee (METC azM/UM 2018-0930).

### Study Population and Recruitment

Individuals with acquired brain injury, potentially with their informal caregivers, who have recently finished their rehabilitation program, were eligible. The rehabilitation center is a healthcare organization providing rehabilitation care with five locations in the South of the Netherlands.

Types of acquired brain injury could be any of the following: traumatic brain injury, cerebrovascular accident, or brain injury as a result of anoxia, infection, or tumor. The inclusion and exclusion criteria are listed in Table 1. Partners, spouses, or significant others who are most closely related to the patient are considered informal caregivers.

**Table 1:** Inclusion and exclusion criteria for patients.

Inclusion criteria	- Individuals with acquired brain injury who completed their rehabilitation treatment (including day rehabilitation).
	- Age 18 years or older
	- Dismissal to their own home after rehabilitation
	- Desire for peer support at home (provided by patient after consultation)
	- Some preserved learning ability
Exclusion criteria	- Discharge to a destination other than home
	- Severe mental health problems, defined as severely increased scores on the Four-Dimensional Symptom Questionnaire (4DSQ) (distress >20, depression >5, anxiety >9, somatization >20)
	- Sufficient professional support at home according to the rehabilitation team
	- Life expectancy <6 months
	- Chronic (>4 months) use of psychotropic medication

Patients will be recruited from June 2019 to September 2020. Annually, around 750 patients with acquired brain injury are admitted to the center. Inpatient as well as outpatient treatments are offered.

By the end of the treatment in the rehabilitation center, the multidisciplinary rehabilitation team discusses the preferable subsequent trajectory after dismissal, including the type of primary or home care for the patient and their informal caregivers. At this point, SMS coaching by a peer supporter is offered to eligible patients (Table 1).

If the patient is willing to participate, a written informed consent is obtained and the patient will be matched with a peer supporter.

### Peer Supporters

Potential peer supporters are recruited both by recommendation of the multidisciplinary rehabilitation team of the rehabilitation center and by patient associations in the area. Important criteria to become an SMS coach are for the peer supporter to: have processed his or her own condition, be a good listener, have sufficient speech possibilities, and be able to travel to the home of the patients. The six-half-day course that the peer supporters follow to become a trained SMS coach is adapted to their cognitive and physical possibilities, including sufficient breaks, frequent repetition, and written information. The three theoretical concepts (exploration, cognitive-behavioral change, and problem solving) are discussed and practiced in role-play with the trainers and the other peer supporters. In the last session, the peer supporters show their skills during a session with a simulation client.

### Intervention

SMS by the peer supporter will be provided during visits at the patient's home. In previous SMS studies, the frequency of visits ranged from 5 to 8 (15). Trained peer supporters will provide SMS to patients that have just finished their rehabilitation treatment and are dismissed home. The patient's rehabilitation physician will offer the SMS by a peer supporter. The intervention starts with exploration. Exploration is a phase in which the peer supporter discusses problems in daily life activities of the patient. Exploration features asking open-ended questions to gather information on this topic and providing insight in the level of psychological burden that the patient experiences with these problems. Cognitive–behavioral change is characterized by challenging irrational thoughts that the patients might have, that are holding them back from performing everyday tasks (e.g., how other people perceive the patient while there are visible limitations, like walking with a walker). Those thoughts are challenged by the peer supporter to check their value and perhaps change these thoughts. For more practical problems, such as outside transportation, problem solving is a possibility for the patient to come up with an action plan. The peer supporter will use a stepwise approach that discusses the advantages and disadvantages of several solutions. This way, the patient can choose the best possible solution for the problem.

The peer supporters are trained to act upon changes in psychosocial well-being of the participants. If specific physical or psychosocial problems appear to be persistent and serious over time, the peer supporter will apply a step-wise approach that starts with verbal administration of the Daily Functioning Thermometer (DFT), a visual analog scale of overall burden (range 0–10). If DFT < 6, the Distress Screener (DS) will be verbally administered. This is a quick-scan instrument to identify potential underlying mental health problems (19). If DS > 3, the Four-Dimensional Symptom Questionnaire (4DSQ) will be used to identify the level of psychological well-being. If at least one of the 4DSQ subscale scores is above the cutoff point (distress > 20, depression > 5, anxiety > 9, somatization > 20) or the peer supporter considers that the psychological situation of the patient is not stable, professional care is necessary. If the patient still has a connection to the rehabilitation center, a healthcare professional at the rehabilitation center will be consulted. Otherwise, the patient will be advised to contact the general practitioner.

### Data Collection

#### Patient Outcomes Pre–post Evaluation

Written questionnaire data will be collected at baseline, i.e., 2–4 weeks after having ended the rehabilitation treatment (T0), immediately after the peer-led SMS intervention (T1), and 6 months later (T2). The degree of self-efficacy, measured by the 12-item Dutch version of the General Self-Efficacy Scale (ALCOS12), consists of three subscales: taking initiative, competence, and perseverance when a setback occurs (20). The *Four-Dimensional Symptom Questionnaire (4DSQ)* is a questionnaire consisting of 50 items that measures psychological well-being in four domains: psychosocial complaints, distress, depression, and anxiety (19). The *MPAQ* is a 16-item questionnaire used to measure the degree of personal autonomy. It consists of three scales: the degree of experienced autonomy, the effort made to achieve autonomy, and dilemmas that doing what is best for the illness might not match a person's valued activities and social roles (21). The *QOLIBRI* measures the health-related quality of life in people with acquired brain injury with 37 items from 6 subscales (22). An overview of the primary and secondary outcome measures is presented in Table 2.

**Table 2:** Patient outcome domains and questionnaires.

**Domain**	**Instrument**	**Measurement**		
		***T0***	***T1***	***T2***
Demographic factors	Specific questions about age, sex, education level, and socioeconomic status	X		
Psychological well-being	Four-Dimensional Symptom Questionnaire (4DSQ)	X	X	X
Self-efficacy	Dutch version of the General Self-Efficacy Scale (ALCOS12)	X	X	X
Brain injury-specific quality of life	Quality of Life after Brain Injury (QOLIBRI)	X	X	X
Personal autonomy	Maastricht Personal Autonomy Questionnaire (MPAQ)	X	X	X
Satisfaction with the SMS coaching	Tailor-made questionnaire		X	

*T0, immediately after dismissal from the rehabilitation center (baseline measurement)*.

*T1, post SMS coaching measurement*.

*T2, follow-up measurement 6 months post SMS coaching*.

Patient characteristics that will be collected are age, sex, subtype of acquired brain injury, time since injury, educational level, income, and whether the patient will receive professional care at home after dismissal.

#### Process Evaluation

To determine the reach of the intervention, frequencies and characteristics of the patients who either accepted or declined the SMS support will be collected. Table 3 provides an overview of the variables collected in the process evaluation. After each visit, peer supporters complete a tailored questionnaire to determine the extent of implementation of the SMS coaching, which is the quantification of the fidelity. Quantitative data through a questionnaire will be gathered on dose delivered, i.e., the number of house visits by peer supporters. Dose received (satisfaction) will be measured with both quantitative methods through the overall grade of the intervention by the patients, whether or not the patients would recommend the intervention to other patients, and the usefulness experienced by the patients, as with qualitative methods through focus groups among professionals, patients, and peer supporters.

**Table 3:** Implementation measures, barriers, and facilitators.

**Domain**	**Stakeholders**	**Data source**	**Method**	
			**Quantitative**	**Qualitative**
Reach: proportion of the intended target population that participated in the intervention	Patients	Descriptive statistics on the number of patients that were given the offer to receive the intervention, compared to patients that refused, dropped out, or completed the intervention	X	
Fidelity: to what extent was the intervention performed as planned	Peer supporters	Checklist that is filled out after every house visit that describes the components of the intervention that were performed. Focus group	X	X
Dose delivered: amount of house visits that took place	Peer supporters	Checklist describing the amount of house visits	X	
Dose received: satisfaction of the patients, peer supporters, and professionals with the intervention	Professionals	Focus group		X
	Patients	- Overall grade of the intervention - Recommendation to others by the patient - Usefulness experienced by the patient - Focus group	X X X	X
	Peer supporters	Focus group		X
Barriers and facilitators: problems that were encountered while implementing the intervention	Peer supporters	- Quantitative data concerning the amount of peer supporters trained compared to peer supporters that signed up - Descriptive information about traits that the succeeded peer supporters possess - Focus group	X	X
	Patients	Focus group		X

Post-intervention, focus groups will be held to gather in-depth qualitative data about the experiences with the intervention. One group will be individuals (i.e., patients and informal caregivers) that received SMS by peer supporters. The second group will be peer supporters themselves. The main goal of both focus groups is to gather in-depth qualitative data of the experiences with the intervention, and the barriers and facilitators that the users may have encountered. In addition, the focus-group discussions may be used to provide deeper insight in the mechanisms of the intervention. This information is gathered in the focus group by asking the participants how the intervention has helped them. A third focus group will be held among professionals that are experts in the field of acquired brain injury; qualitative data will be collected on the cooperation between peer supporters and professionals at the rehabilitation center, as well as on the implementation in clinical practice.

#### Implementation

To optimize implementation, an evaluation of an implementation pilot phase of 3 months will be held at the rehabilitation center. The peer supporter will be embedded in usual care at the rehabilitation center. This phase will take place after the recruitment of participants in order to discover how implementation of the peer-led SMS intervention can be improved. This will be evaluated through a group interview and a questionnaire among healthcare professionals and peer supporters. Topics of this group interview will include their experiences with this implementation, the implications for usual care, and possibilities for improvement.

#### Dissemination

Relevant stakeholders in rehabilitation after acquired brain injury (i.e., health insurance financiers, volunteer organizations, and content specialists) will be gathered in a workshop. The goal of this workshop is to discuss what is necessary for a successful implementation of cooperation between healthcare professionals and peer supporters. With this information, the target population for the peer-led intervention can be disseminated to other regions and people with acquired brain injury who do not necessarily receive specialized rehabilitation.

After completion of the study, the data will be publically accessible for further research and verification from a data repository platform (Dataverse NL).

### Statistical Analysis

The mixed-method design of this implementation study aims to triangulate the findings of quantitative and qualitative analyses (23). One aspect of the quantitative analyses is the evaluation of patient outcomes with the degree of self-efficacy as the primary outcome. As a guideline, a required sample size of 90 participants was calculated based on an effect size of 0.3 (24, 25), an α of 0.05, and a statistical power (1-β) of 0.80. Furthermore, the qualitative data collection in two focus groups will be held among eight participants for both groups.

Standard descriptive statistics will be used to present the data concerning the participants, dropouts, losses-to-follow-up, and their characteristics. Comparisons of the mean values in patient-reported outcomes over time will be analyzed using paired *t*-tests, or non-parametric tests in case of a skewed distribution and, if possible, multilevel repeated analyses. Furthermore, we will conduct subgroup analyses to study whether the change in self-efficacy over time differs according to demographic characteristics, such as age, sex, educational level, and socioeconomic status, or the “dose” of peer-led SMS received by the patient.

Equally important, with our interest in matters of implementation, there is a qualitative part in the study. Within the focus groups, we will seek for new information until saturation is reached. The qualitative data will be coded and analyzed by two individuals.

## Discussion

We present the protocol of a mixed-method study to evaluate the implementation of a peer-led self-management intervention following acquired brain injury rehabilitation. In a pre–post evaluation, quantitative changes in patient outcome pre–post intervention are complemented with qualitatively derived patient experiences post-intervention. To evaluate the process of implementation, registrations during the intervention and post-intervention focus-group discussions with patients, informal caregivers, peer supporters, and health professionals are used to investigate the extent of implementation and the barriers and potential facilitators. A workshop with relevant stakeholders will be held to gather recommendations for successful dissemination of the peer-led intervention.

Self-management and peer support by volunteers are the core components of the current SMS intervention. Self-management interventions have proven effective in patients with chronic diseases, and various approaches have been described (9–11). For improving active coping and the patient's self-efficacy and mastery, these interventions have shown to be supportive for patients with an acquired brain injury (10, 11, 13, 14). Patients with acquired brain injury that demonstrate active coping mechanisms have shown more positive results regarding health-related quality of life and participation after following a self-management intervention (26). Furthermore, it is increasingly common to involve peer supporters in the treatment of mental health problems. Only few studies evaluated the implementation of peer support programs for people with an acquired brain injury. These show promising results, and patients report positive experiences with peer supporters (7, 17, 18, 27). Furthermore, support provided by volunteers is a low-cost method.

The results of the previous SMS studies in the primary care setting were promising. Unfortunately, the implementation of SMS progressed slowly and eventually stagnated due to the high workload of healthcare professionals and the disease-oriented context that they work in (14, 15). As mentioned, the adaptation phase after brain injury is challenging for many patients and their informal caregivers, and there are unmet care needs (4). Introducing a self-management intervention in this phase is recommended by both the patients and their informal caregivers (28, 29). Peer supporters are believed to be of added value in addition to professional care, because they recognize and can relate to the patients' needs more by experience (4). Both the SMS intervention itself and the unique qualities of peer supporters who provide the intervention have shown to be promising components of support programs and are therefore believed to constitute a strong base for implementation (6, 7, 10, 17, 18).

In summary, this paper describes the design of a study on the barriers and facilitators related to the implementation of a home-based, peer-led self-management intervention for patients with an acquired brain injury. We will quantitatively and qualitatively evaluate the change in relevant patient outcomes pre- and post-intervention. Equally important, quantitative registrations and qualitative research among patients, their informal caregivers, peer supporters, and respective professionals will provide insight into the barriers and facilitators related to the implementation of the intervention. Enrollment of patients and informal caregivers has started in June 2019 and will continue until September 2020. Currently, we experience substantial problems in patient recruitment due to the corona pandemic.

## Ethics Statement

The studies involving human participants were reviewed and approved by METC aZM/UM. The patients/participants provided their written informed consent to participate in this study.

## Author Contributions

MB-D has drafted the manuscript. SS designed the study and substantively revised the manuscript. JV designed the study and substantively revised the manuscript. HB made substantial contributions to the conception, designed the study, and substantively revised the manuscript. JE made substantial contributions to the conception, designed the study, and substantively revised the manuscript. All authors have read and approved the manuscript.

## Conflict of Interest

The authors declare that the research was conducted in the absence of any commercial or financial relationships that could be construed as a potential conflict of interest.

## Publisher's Note

All claims expressed in this article are solely those of the authors and do not necessarily represent those of their affiliated organizations, or those of the publisher, the editors and the reviewers. Any product that may be evaluated in this article, or claim that may be made by its manufacturer, is not guaranteed or endorsed by the publisher.
